# The band count imprecision – a Croatian multicentric pilot study

**DOI:** 10.11613/BM.2024.020803

**Published:** 2024-06-15

**Authors:** Vanja Radišić Biljak, Višnja Jureša, Valentina Vidranski, Ivana Vuga, Franciska Tomić, Fran Smaić, Martina Horvat, Branka Krešić, Brankica Šimac, Ivana Lapić

**Affiliations:** 1 Department of Medical Laboratory Diagnostics, University Hospital Sveti Duh, Zagreb, Croatia; 2 Department of Sport and Exercise Medicine, University of Zagreb, Faculty of Kinesiology, Zagreb, Croatia; 3 Working Group for Laboratory Hematology of the Croatian Society of Medical Biochemistry and Laboratory Medicine; 4 Department of Medical Biochemistry, General County Hospital Vinkovci, Vinkovci, Croatia; 5 Department of Oncology and Nuclear Medicine, Sestre milosrdnice University Hospital Center, Zagreb, Croatia; 6 Department of Clinical Chemistry, Sestre milosrdnice University Hospital Center, Zagreb, Croatia; 7 Department of Laboratory Diagnostics, General Hospital Dr. Josip Benčević, Slavonski Brod, Croatia; 8 Department of Medical Laboratory Diagnostics, University Hospital of Split, Split, Croatia; 9 Clinical Department of Laboratory Diagnostics, University Hospital Dubrava, Zagreb, Croatia; 10 Department of Laboratory Diagnostics, University Hospital Centre Zagreb, Zagreb, Croatia; 11 University of Zagreb, Faculty of Pharmacy and Biochemistry, Zagreb, Croatia

**Keywords:** neutrophils, observer variation, hematology

## Abstract

**Introduction:**

Due to high inter-observer variability the 2015 International Council for Standardization in Haematology (ICSH) recommendations state to count band neutrophils as segmented neutrophils in the white blood cell (WBC) differential. However, the inclusion of bands as a separate cell entity within the WBC differential is still widely used in hematology laboratories in Croatia. The aim of this multicentric study was to assess the degree of inter-observer variability in enumerating band neutrophils within the WBC differential among Croatian laboratories.

**Materials and methods:**

Seven large Croatian hospital laboratories from different parts of the country participated in the study. In each of 7 participating laboratories, one blood smear, that was flagged by the analyzer as possibly having bands, was evaluated by all personnel participating in the analysis of hematology samples. Between-observer manual smear reproducibility was expressed as coefficient of variation (CV) and calculated using the following formula: CV (%) = (standard deviation (SD)/mean value) x 100%.

**Results:**

The CVs (%) and relative band neutrophil counts in participating laboratories were as follows: 15.4% (16-24), 19.2% (16-32), 19.5% (17-40), 21.1% (17-44), 35.0% (8-26), 51.9% (3-29), and remarkably high 62.4% (12-59). For segmented neutrophils CVs were lower, ranging from 7.4% to 32.2%. The CVs did not correlate with the number of staff members in each hospital (P = 0.293).

**Conclusions:**

This study revealed very high variability in enumerating band neutrophil count in the blood smear differential among all participants, thus prompting a need for action on a national level.

## Graphical abstract

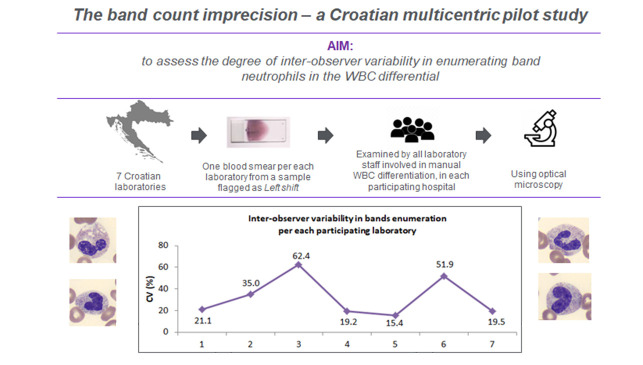

## Highlights

• Band counting is unreliable practice due to the high inter-observer variability

• 2015 International Council for Standardization in Haematology guidelines recommend to count band neutrophils as segmented neutrophils

• The inclusion of bands within the white blood cell differential is still used in Croatia

• There is a very high variability in enumerating bands among Croatian laboratories

• There is a need for national recommendations that will endorse ICSH guidelines

## Introduction

Band neutrophils are defined as granulocytes with nonsegmented nucleous or rudimentary lobes connected by a thick band. This term is related to the penultimate maturing stage in the process of granulocytopoiesis, preceeding segmented neutrophil granulocytes. Elevated band neutrophils count is nonspecific and can be found in a variety of pathophysiological conditions that trigger release of neutrophil reserves from the bone marrow, the most common being bacterial infections, inflammation, postsurgical complications or injuries. Due to lack of consistent definition and hence the ambiguity in morphological characterization, distinguishment between segmented and band neutrophils is prone to observer’s subjectivity (*1*). Consequently, large evidence shows that neutrophil bands counting and reporting of bands count is unreliable practice due to high inter-observer variability (*1*, *2*). Therefore, the recommendations of the International Council of Standardization in Haematology (ISCH) issued in 2015 state that band neutrophils should be counted as segmented neutrophils in the white blood cell (WBC) differential. In spite of that, the inclusion of bands as a separate cell entity within the WBC differential is still widely used in hematology laboratories in Croatia (personal communication).

In 2018 the Working Group for Laboratory Hematology (WGLH) of the Croatian Society of Medical Biochemistry and Laboratory Medicine was established, with the aim to standardize and harmonize practices in laboratory hematology in Croatia. Previously, a study to quantify the degree of the inter-observer variability of the band neutrophils counting in manual WBC differential blood count was conducted in one mid-sized laboratory in Croatia (*2*). The results of the study were very disappointing, with CVs ranging from 53.6% up to remarkably high 100% (*2*). However, the respective study reflects only the practices of one medium-sized clinical laboratory. Therefore, for a more objective insight into current practices in differentiating band from segmented neutrophils, it was essential to conduct a similar but wider study that would include several laboratories from different parts of the country. Hereby we present the results of a multicentric study that was conducted with the aim to assess the degree of inter-observer variability in enumerating band neutrophils in the WBC differential among Croatian laboratories.

## Materials and methods

In November and December 2021, seven large Croatian hospital laboratories from different parts of the country participated in the study. Table 1 lists the participating institutions, the staff involved in the study, and equipment used in the respective hematology laboratories.

**Table 1:** Characteristics of participating laboratories

**Number**	**Hospital name**	**Department**	**City**	**Number of staff members included in the study**	**Level of education**	**Type of tubes**	**Hematology analyzer**	**Optical microscope**
1	University Hospital Sveti Duh	Department of Medical Laboratory Diagnostics	Zagreb	16	four specialists in laboratory medicine, two residents in laboratory medicine, two bachelor degree laboratory technicians, and eight laboratory technicians	BD K2EDTA (BD-Plymouth, UK)	Siemens Advia 2120i (Siemens Healthineers, Marburg, Germany)	Olympus BX53F2 (Olympus, Tokyo, Japan)
2	General County Hospital Vinkovci	Department of Medical Biochemistry	Vinkovci	20	three specialists in laboratory medicine, two residents in laboratory medicine, six bachelor degree laboratory technicians, and nine laboratory technicians	BD K3EDTA (BD, Plymouth, UK)	Siemens Advia 2120i (Siemens Healthineers, Marburg, Germany), Sysmex XN 550 (Sysmex Corporation, Kobe, Japan)	Olympus BX 50F-4 (Olympus, Tokyo, Japan)
3	Sestre milosrdnice University Hospital Center	Department of Oncology and Nuclear Medicine	Zagreb	7	two residents in laboratory medicine, one molecular biologist, one bachelor degree laboratory technician and three laboratory technicians	Greiner K3EDTA (Greiner Bio-One GmbH, Kremsmünster, Austria)	Sysmex XN 330 (Sysmex Corporation, Kobe, Japan)	Laboval (Zeiss, Oberkochen, Germany)
4	Sestre milosrdnice University Hospital Center	Department of Clinical Chemistry	Zagreb	11	three specialists in laboratory medicine, one resident in laboratory medicine, three bachelor degree laboratory technicians, four laboratory technicians	Greiner K3EDTA (Greiner Bio-One GmbH, Kremsmünster, Austria)	Sysmex XN 1000 (Sysmex Corporation, Kobe, Japan)	Olympus BX43 (Olympus, Tokyo, Japan)
5	General Hospital Dr. Josip Benčević	Department of Laboratory Diagnostics	Slavonski Brod	5	two specialists in laboratory medicine, one bachelor degree laboratory technicians, two laboratory technicians	BD K3EDTA (BD, Plymouth, UK)	Sysmex XN 1000 (Sysmex Corporation, Kobe, Japan)	Olympus CX43RF (Olympus, Tokyo, Japan)
6	University Hospital Split	Department of Medical Laboratory Diagnostics	Split	23	two specialists in laboratory medicine, two residents in laboratory medicine, four bachelor degree laboratory technicians, fifteen laboratory technicians	BD K3EDTA (BD, Plymouth, UK)	Siemens Advia 2120i (Siemens Healthineers, Marburg, Germany)	Olympus CX40 (Olympus, Tokyo, Japan)
7	University Hospital Dubrava	Clinical Department of Laboratory Diagnostics	Zagreb	12	two specialists in laboratory medicine, five bachelor degree laboratory technicians, five laboratory technicians	BD K3EDTA (BD, Plymouth, UK)	Siemens Advia 2120i (Siemens Healthineers, Marburg, Germany)	Olympus CX41 (Tokyo, Japan)

### Methods

In each hospital separately, one whole blood sample drawn in tubes containing potassium ethylenediaminetetraacetic acid as the anticoagulant (listed in Table 1), previously analyzed on an automated hematology analyzer (listed in Table 1) was selected. A sample flagged by the analyzer as possibly containing band neutrophils („left-shift“) was chosen. Blood smears were prepared and stained manually by using the May Grünwald- Giemsa technique. The selected blood smear was examined by all staff members (the level of education listed in the Table 1) involved in manual WBC differentiation, in each participating hospital. The whole process was identical as in the previously published study (*2*). Namely, all staff members performed one manual differential count (by counting 100 cells) by using optical microscope (Table 1), thus simulating the usual routine laboratory process. Neither of the staff members had insight into the results of the other examiners, meaning that the whole process was single-blinded.

### Statistical analysis

Data was stored in Microsoft Excel 2010 program (Microsoft, Microsoft Excel, Redmond, USA). Relative band counts were presented as medians with the appropriate ranges (min–max). Between-observer manual smear reproducibility was expressed as coefficient of variation (CV) and calculated using the following formula: CV (%) = (standard deviation (SD) / mean value) x 100%. The correlation of CVs with the number of observers was assessed by calculating Spearman’s rank correlation coefficient. All statistical analyses were performed in MedCalc® v22.016 statistical software (MedCalc Software, Ostend, Belgium).

## Results

The conducted study revealed very high variability in band cells enumeration between the observers in the majority of participating laboratories. The CVs (%) and band neutrophil ranges were as follows: 15.4% (16-24), 19.2% (16-32), 19.5% (17-40), 21.1% (17-44), 35.0% (8-26), 51.9% (3-29), and remarkably high 62.4% (12-59) (Table 2).

**Table 2:** A multicentric (N = 7) inter-observer variability in segmented neutrophils, band neutrophils, and total (segmented + bands) neutrophils differential count

**CVs %**	**Participating laboratories**						
Cells median (range)	1	2	3	4	5	6	7
Band neutrophils	21.1 28 (17-44)	35.0 15 (8-26)	62.4 26 (12-59)	19.2 23 (16-32)	15.4 21 (16-24)	51.9 13 (3-29)	19.5 32 (17-40)
Segmented neutrophils	15.9 51 (34-67)	14.2 68 (52-77)	32.2 55 (21-68)	9.9 67 (52-75)	9.6 68 (62-78)	7.4 79 (64-89)	20.9 43 (37-70)
Segmented + band neutrophils	5.3 80 (72-86)	7.7 82 (78-89)	5.6 80 (74-88)	3.0 90 (84-94)	3.9 90 (86-94)	5.0 92 (79-99)	7.0 75 (68-87)
CV % - coefficent of variation, expressed in percentages.							

The CVs did not correlate with the number of observers in each hospital (P = 0.293). For segmented neutrophils CVs were lower, ranging from 7.4% to 32.2%. When the number of bands was added to the number of segmented neutrophils, the CVs remarkably decreased, ranging from merely 3.0% to a maximum of 7.7%. Two of the participating hospitals revealed slightly higher imprecision in neutrophil count enumeration compared to others.

## Discussion

The present study revealed significant inter-observer variability in enumerating band neutrophils and heterogeneity in their distinguishment from segmented neutrophils.

The overall high inter-observer CVs are in accordance with the previously published initial excercise and some international studies, once again pointing out to the ambiguity of accurate band neutrophils enumeration (*2*-*4*). These technical problems, together with the lack of disease specificity and diagnostically low relevance of elevated band neutrophils count, limit reporting of band neutrophils as a separate cell entity. Therefore, it is reasonable for laboratories to follow the recommendation issued by ICSH and count band as segmented neutrophils in the WBC differential (*5*). This is further supported by the fact that such approach significantly decreases inter-observers’ CV, as evidenced herein. In fact, an increase in the proportion of band neutrophils is usually accompanied by an increase in the absolute number of neutrophils, hence the overall neutrophil count is an equally reliable parameter to reflect these changes.

The present study has some limitation. Firstly, there was no uniformity in the criteria for defining band neutrophils among the participating laboratories. Additionally, each participating laboratory analyzed only one separate smear with a different WBC differential. It can be assumed that the distribution of results would be more uniform if all staff involved in peripheral blood smear differentiation in all centers performed WBC differential using the same blood smear. However, a general problem which all laboratories are facing is lack of skilled and experienced technicians. Hence, we can presume that personnel turnover also contributed to the observed substantial variability. However, as in the original study published by the Radišić Biljak *et al.,* while simulating everyday routine work, we were able to assess the real-life performance of this manual technique, understand the size of the uncertainty of the reported results and compare it between different sites spread all over the country (*2*).

The obtained results revealing very high variability in enumerating band neutrophil count in the blood smear differential among all participants, indicate a need for harmonization of practices on a national level. Based on the limited clinical utility of the band count and the recommendations to include them within the relative neutrophil count, a systematic approach should be designed with the ultimate aim to abandon reporting band cells count as a separate entity (*5*). This should be inevitably done in agreement with key clinicians, after thorough explanation of the rationale behind it. Laboratory rules for manual smear review should be revised and the flags indicating the presence of bands should be excluded as a review criteria. Laboratory staff involved in manual smear review should be instructed about these changes and the ongoing practice that bands should be counted as neutrophil granulocytes. In situations when an unusually high number of bands is seen in the blood film, addition of an appropriate comment should be considered, which is in accordance with ICSH recommendations (*5*).

As confirmed in the present study, it would be valuable to spread this practice among all Croatian medical laboratories in order to improve harmonization in the field. Nevertheless, a national consensus driven by appropriate future national recommendations written by the WGLH is required.

## Data Availability

The data generated and analyzed in the presented study are available from the corresponding author on request.
